# Psychodermatologic Dimensions of Psoriasis: Perceived Burden, Help-Seeking Behavior and Openness to Supportive Care

**DOI:** 10.3390/jcm15155984

**Published:** 2026-07-31

**Authors:** Bogdan Marian Tarcau, Dan Vata, Ioana Adriana Popescu, Doinita Temelie Olinici, Angelica Postu, Ioana Alina Halip, Dragos Florin Gheuca Solovastru, Madalina Mocanu, Marcel Alexandru Gaina, Laura Gheuca Solovastru

**Affiliations:** 1 Grigore T. Popa University of Medicine and Pharmacy Iasi, 700115 Iasi, Romania; bogdan.tarcau@umfiasi.ro (B.M.T.); doinitzaganceanu@yahoo.com (D.T.O.); alinaioanahalip@gmail.com (I.A.H.); dragosflorin13@yahoo.com (D.F.G.S.); drmadalinamocanu@yahoo.com (M.M.); marcel-alexandru_t_gaina@d.umfiasi.ro (M.A.G.); lsolovastru13@yahoo.com (L.G.S.); 2 Department of Dermatology, Grigore T. Popa University of Medicine and Pharmacy Iasi, 700115 Iasi, Romania; 3 Department of Morpho-Functional Sciences II, Grigore T. Popa University of Medicine and Pharmacy Iasi, 700115 Iasi, Romania; 4 Department of Music Therapy, Berlin University of the Arts, 10623 Berlin, Germany; a.postu@udk-berlin.de; 5 Department of Psychiatry, Grigore T. Popa University of Medicine and Pharmacy Iasi, 700115 Iasi, Romania

**Keywords:** psoriasis, psychodermatology, stress, music therapy, help-seeking behavior, stigma, cross-sectional study

## Abstract

**Background/Objectives**: Psoriasis is a chronic immune-mediated dermatosis with an important psychodermatologic burden, in which psychological stress, perceived disease impact, stigma, and attitudes toward mental health care may influence patient support needs. Although psychological and psychiatric interventions are increasingly discussed in dermatology, patient openness to different forms of supportive care, including music therapy, remains insufficiently characterized. This study aimed to characterize psychodermatologic burden and support-related attitudes among patients with psoriasis, with particular emphasis on openness to psychological counseling, psychiatric care, and music therapy-based support, and to explore the factors associated with these attitudes. **Methods**: In this single-center cross-sectional study, 102 adults with cutaneous or nail psoriasis completed an original self-administered questionnaire between July 2023 and January 2025. **Results**: Most participants perceived stress as related to psoriasis onset or worsening (86.3%) and considered dermatologist guidance toward psychological or psychiatric support beneficial (80.4%). Psychological counseling was the most accepted supportive intervention (75.5%), followed by music therapy (54.9%) and psychiatric consultation (48.0%), while previous psychological or psychiatric care was reported by 22.5%. Willingness to seek psychological support was independently associated with perceived psoriasis impact, whereas willingness to seek psychiatric support was associated with perceived stress impact. Awareness of music therapy was associated with higher educational level. Willingness to try music therapy was associated with high perceived stress impact, higher educational level, music listening, and awareness of music therapy. **Conclusions**: Patients with psoriasis frequently perceive stress as relevant to their disease and show variable openness to supportive care. These findings support an individualized psychodermatologic approach in which dermatologists may help identify patients receptive to psychological, psychiatric, or music therapy-based interventions.

## 1. Introduction

Psoriasis (PsO) is a chronic, immune-mediated inflammatory dermatosis affecting a substantial proportion of the global population, with reported prevalence rates ranging from 0.91% to 8.5% [1]. Its development and recurrence are influenced by a complex interplay of genetic, epigenetic, environmental, and lifestyle-related factors [2]. Among the lifestyle-related factors, psychological stress has an important role, influencing both disease onset in predisposed individuals and the occurrence of exacerbations [3,4]. Psychological stress may be understood within the transactional framework of Lazarus and Folkman, in which stress depends not only on external demands, but also on the individual’s appraisal of those demands and perceived coping resources [5]. Daily stressors have been associated with increased itch and greater PsO severity, particularly among individuals with a higher tendency toward excessive worry [6,7]. Conversely, living with a chronic, visible, and symptomatic disease such as PsO may itself contribute to psychological distress through social stigma, impaired body image, sexual dysfunction, anxiety, and depression, with a substantial negative impact on quality of life [8,9,10,11,12]. Taken together, these pathways support a bidirectional relationship between psychological stress and PsO.

Psychodermatology is an emerging field within dermatology that supports the management of skin diseases through psychological and psychiatric interventions aimed at reducing stress and improving maladaptive thoughts and behaviors [13]. Various psychological interventions, including cognitive behavioral therapy, hypnosis, meditation, guided imagery, and music therapy, have been explored as adjunctive strategies in the management of PsO [14,15,16].

Although the psychodermatologic burden of PsO is well documented, less is known about how patients translate this burden into actual help-seeking behavior or acceptance of supportive care. Previous studies have reported substantial psychiatric morbidity among patients with PsO, particularly depression and anxiety, but the use of psychological and psychiatric services appears considerably lower than the potential need for such care [9,11]. In a questionnaire-based Romanian PsO cohort, 23% of patients reported having consulted a psychologist and 7% a psychiatrist, while a prospective screening study found that 23.9% of patients accepted psychiatric treatment after it had been recommended [17,18]. These findings suggest a possible discrepancy between psychological burden, expressed openness to support, and actual use of mental health services [17,18].

Help-seeking decisions may be influenced by perceived need, stigma, previous experience with care, treatment preferences, and the perceived usefulness of professional support [17,18,19,20,21]. Mental health-related stigma may reduce active help-seeking, while psychological treatment is generally viewed more favorably than pharmacological or psychiatric treatment [18,19,20].

Music therapy has received less attention in PsO than other psychological or behavioral interventions, but it may represent a potentially acceptable complementary approach for stress reduction [22]. Music therapy refers to the structured, clinical use of music-based interventions to support therapeutic goals, such as stress reduction, emotional regulation, relaxation, and coping, and should be distinguished from informal music listening [23]. Music-based interventions have been explored in dermatology mainly in relation to anxiety, pain, procedural distress, and relaxation, while earlier studies also suggested possible benefits in inflammatory dermatoses, including PsO [22,24]. Complementary approaches have been reported in approximately 56.5–62% of patients with PsO, indicating interest in non-pharmacological forms of support [25,26,27]. Given the limited evidence regarding patient acceptability of music therapy in PsO, the present study selected music therapy as the predefined complementary intervention of interest within a broader research program. As music is familiar, non-invasive, and often already used by patients for relaxation, assessing awareness of and openness to music therapy may provide useful information for developing patient-centered psychodermatologic interventions.

Evidence remains limited regarding patients’ relative openness to different forms of supportive care. In particular, it is unclear whether patients view psychological counseling, psychiatric consultation, and music therapy-based support differently, and which patient-reported, sociodemographic, care-related, and music-related factors are associated with acceptance of each option. The primary aim of this study was to characterize patients’ openness to three forms of supportive care: psychological counseling, psychiatric consultation, and music therapy-based support. Secondary exploratory objectives were to describe perceived PsO impact, perceived stress impact, patients’ views on the stress–psoriasis relationship, previous use of psychological or psychiatric care, and dermatologist-related guidance, and to examine the factors associated with openness to each form of support.

## 2. Materials and Methods

This single-center, cross-sectional study included 102 patients with PsO who completed an original self-administered questionnaire during visits to the Dermatology Clinic of “Saint Spiridon” County Emergency Clinical Hospital, Iasi, Romania, between July 2023 and January 2025. Inpatients or outpatients were screened for eligibility by a member of the research team. Patients were recruited using a consecutive sampling approach during routine clinical visits. Eligibility required a dermatologist-confirmed diagnosis of cutaneous or nail PsO and age ≥ 18 years. Eligible patients received verbal and written information about the study and were invited to participate voluntarily. After providing written informed consent, participants completed the self-administered questionnaire once, during the clinical visit, without receiving financial or other incentives. The questionnaire consisted of closed-ended items, including dichotomous and multiple-choice questions, as well as numeric rating scales.

The questionnaire was specifically developed for the purposes of this study and consisted of original items designed by the authors. The questionnaire was developed to operationalize the predefined domains of the study, including sex, age, educational level, clinical type of PsO, perceived PsO and stress impact, beliefs regarding the relationship between stress and PsO, dermatologist-related guidance, previous use of psychological or psychiatric care, attitudes toward psychological and psychiatric support, and awareness of and willingness to try music therapy. Because these items addressed distinct descriptive and attitudinal domains, the questionnaire was not designed as a unidimensional psychometric scale. No formal content-validity assessment, construct validation, reliability testing, or pilot testing was performed before administration. Accordingly, the questionnaire should be considered an exploratory study-specific survey instrument rather than a validated psychometric measure. No prospective follow-up assessments were performed. The questionnaire was administered in Romanian, and the English translation of the full questionnaire is provided in the Supplementary Materials as Supplementary File S1: Study questionnaire.

Music therapy was selected a priori as the complementary intervention of interest because the present survey represented a pre-implementation component of a broader doctoral research project aimed at developing and subsequently evaluating a structured music therapy intervention for patients with psoriasis in the local clinical setting.

No a priori sample size calculation was performed. The sample size was determined by the number of eligible patients who completed the questionnaire during the predefined recruitment period; therefore, the analyses were considered exploratory.

The study was conducted in accordance with the principles of the Declaration of Helsinki and applicable Romanian regulations regarding research ethics and personal data protection. Ethical approval was obtained from the Ethics Committee of “Grigore T. Popa” University of Medicine and Pharmacy, Iasi, Romania (Project identification code 293; approved on 14 April 2023). Patients were informed about the study objectives, data confidentiality, and the voluntary nature of participation, and provided written informed consent before completing the questionnaire. All data were anonymized prior to analysis, and no identifying information was used.

Perceived impact of PsO on daily life, perceived impact of psychological stress on daily life, and perceived dermatologist involvement in stress-related care were assessed using 0-to-10 numerical rating scales. The categorization of perceived stress impact into low (≤5) and high (>5) groups was used pragmatically for exploratory group comparisons and was not intended to represent a validated clinical cutoff. To reduce the loss of information associated with dichotomization, the continuous perceived stress impact score was also retained in the relevant non-parametric and multivariable analyses. The single-item numerical rating scale was intended to capture the patient-reported perceived impact of psychological stress on daily life over the previous three months. It was included as a brief, clinically feasible exploratory measure and was not intended to replace a validated multidimensional stress instrument. Educational level was dichotomized into low (≤10 classes or high school diploma) and high (university, master’s, or doctoral studies). Attitudes toward psychological and psychiatric support were collected as four-category responses. For logistic regression analyses, these variables were dichotomized into acceptance versus non-acceptance, with the response “It seems like a good idea if it can help me” coded as acceptance and all other response options, including shame-related responses, fear of social judgment, and explicit refusal (“I do not need it”), coded as non-acceptance. Stigma-related responses were additionally analyzed separately as barriers to mental health support.

Descriptive data were presented as frequencies and percentages for categorical variables and as mean ± standard deviation (SD) or median [interquartile range (IQR)] for continuous variables, as appropriate. Chi-square or Fisher’s exact tests were used for categorical comparisons, with odds ratios (OR) and 95% confidence intervals (CI) reported as effect measures. Sex-related comparisons of continuous variables were assessed using Student’s *t*-test and summarized with Cohen’s d. The distributions of continuous variables were assessed using the Shapiro–Wilk test and visual inspection of histograms and Q–Q plots. Given the approximately balanced group sizes and the absence of significant differences in variances between women and men, Student’s *t*-test was retained as the primary analysis for sex-related comparisons. Mann–Whitney U tests were additionally performed as sensitivity analyses for the numerical rating variables. Comparisons of perceived stress and PsO burden according to help-seeking outcomes were performed using Mann–Whitney U tests, with effect size expressed as r. Multivariable binary logistic regression models were constructed to assess factors independently associated with the perceived relationship between stress and PsO, willingness to seek psychological support, willingness to seek psychiatric support, perceived benefit of guidance by the dermatologist toward a psychologist/psychiatrist, previous psychological counseling or psychiatric treatment, awareness of music therapy, and willingness to try music therapy. For willingness to try music therapy, separate clinical-demographic and music-related models were constructed. For the clinical-demographic model assessing willingness to try music therapy, perceived stress impact was entered as a dichotomous variable, using the predefined high-stress threshold (>5 versus ≤5). Regression coefficients (β), standard errors (SE), ORs, 95% CIs, and *p*-values were reported for all multivariable models. The dataset was complete for all variables included in the statistical analyses; therefore, no imputation of missing data was required. Statistical analyses were performed using SPSS version 22.0 (IBM Corp., Armonk, NY, USA), and a two-sided *p*-value < 0.05 was considered statistically significant.

## 3. Results

### 3.1. Descriptive Results

The sociodemographic and clinical characteristics of the study population are presented in Table 1.

Table 2 presents the patients’ self-rated impact of psoriasis and psychological stress on daily life over the previous 3 months, assessed on a 0-to-10 scale, as well as their perception of the relationship between stress and the onset or aggravation of psoriasis.

Table 3 presents the extent to which patients felt that psychological stress had been addressed by their dermatologist, as well as their perceived benefit of referral to psychological or psychiatric support and their previous use of such services.

Table 4 presents patients’ attitudes toward psychological counseling, psychiatric care, and music therapy, including awareness of music therapy and the main reasons for refusing this intervention, based on preset answers.

### 3.2. Sex-Related Comparisons

Table 5 presents the sex-based comparison of the main dichotomous psychodermatologic variables evaluated in the study.

Shapiro–Wilk testing indicated deviations from normality for age (W = 0.962, *p* = 0.005), perceived psoriasis impact (W = 0.950, *p* < 0.001), and perceived dermatologist involvement in stress-related care (W = 0.862, *p* < 0.001), whereas perceived stress impact did not significantly deviate from normality (W = 0.975, *p* = 0.053).

Sex-related differences in continuous variables are presented in Table 6.

Sensitivity analyses using the Mann–Whitney U test showed differences in the same direction as the primary parametric analyses.

### 3.3. Stress, Perceived Burden and Help-Seeking Behavior

Table 7 presents the multivariable logistic regression model constructed to identify factors independently associated with patients’ perceived relationship between stress and psoriasis.

Help-seeking and support-related outcomes according to stress level are presented in Table 8.

**Figure 1:**
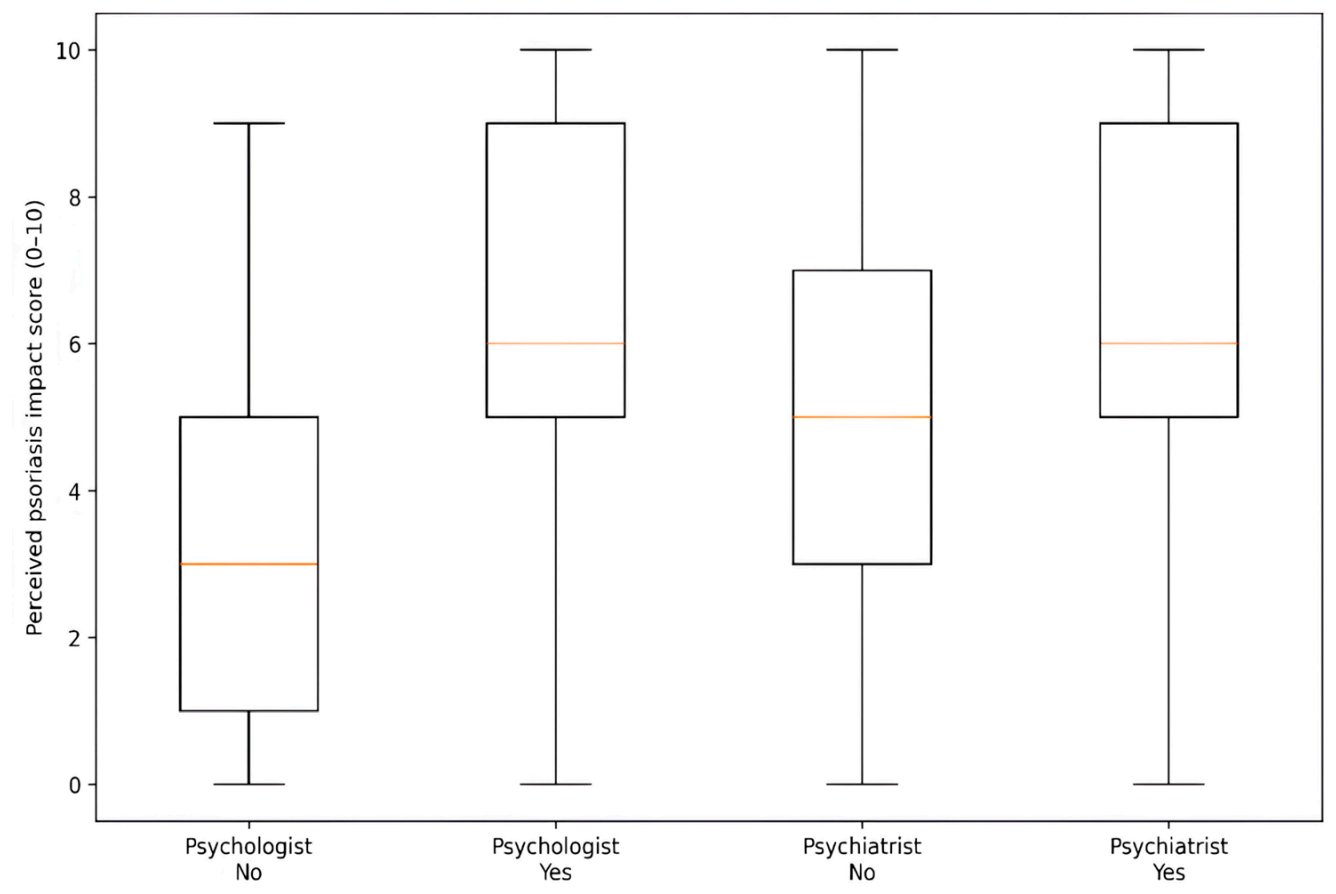
Perceived psoriasis impact according to willingness to seek psychological or psychiatric support. Boxes represent the interquartile range, the horizontal orange line within each box represents the median, and the whiskers extend to the most extreme values within 1.5 times the interquartile range.

**Figure 2:**
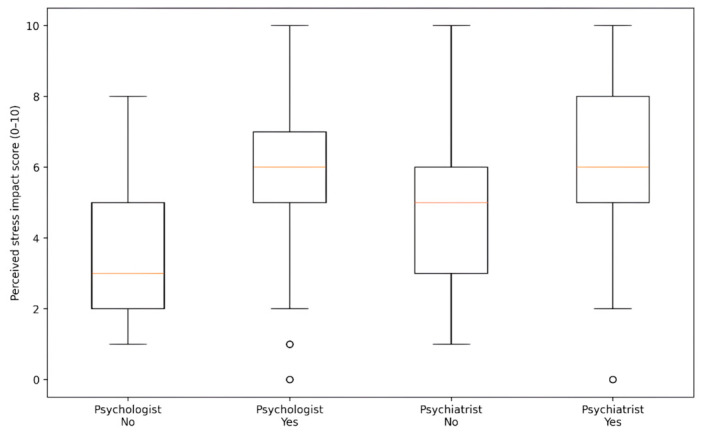
Perceived stress impact according to willingness to seek psychological or psychiatric support. Boxes represent the interquartile range, the horizontal orange line within each box represents the median, and the whiskers extend to the most extreme values within 1.5 times the interquartile range.

Since dichotomization of stress scores may obscure gradations in perceived burden, additional analyses were performed using continuous stress- and psoriasis-impact scores. These results are presented in Table 9 and Figure 1 and Figure 2.

**Figure 3:**
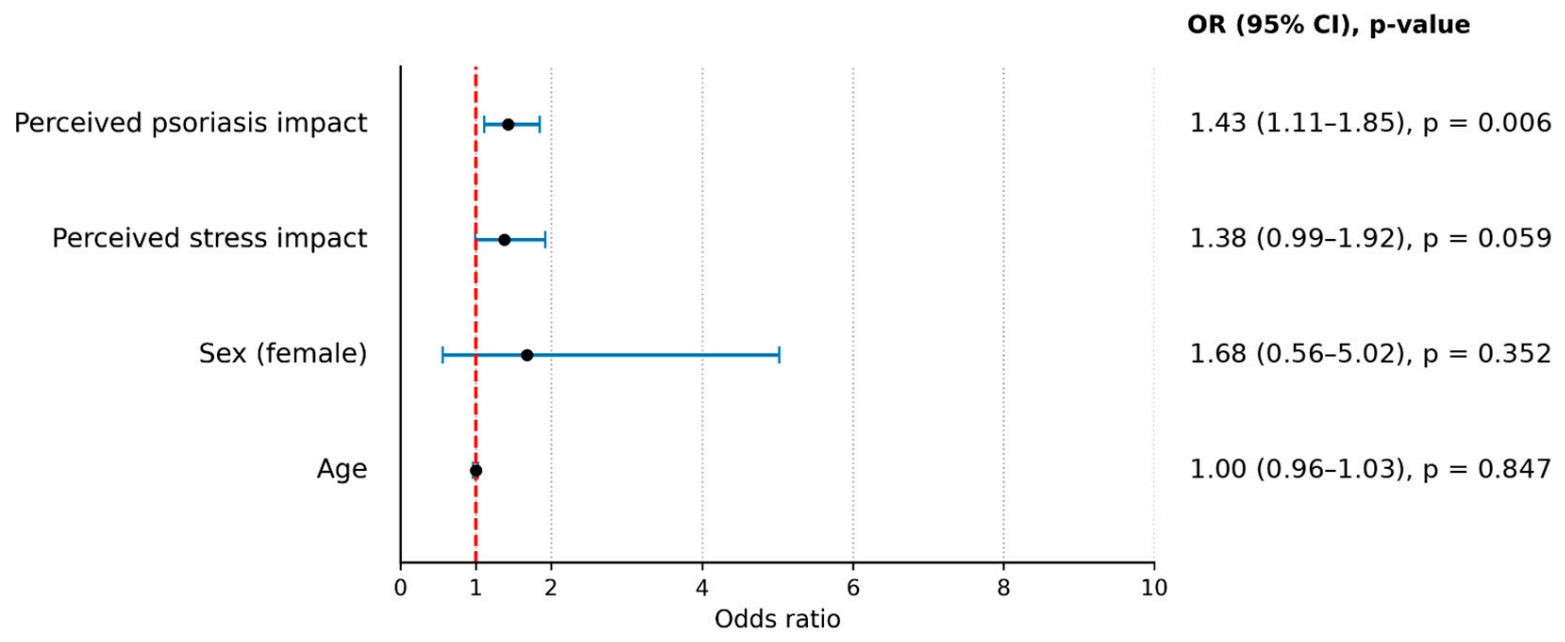
Forest plot showing adjusted odds ratios and 95% confidence intervals for factors associated with willingness to seek psychological support. Black dots represent the adjusted odds ratios, blue horizontal lines represent the corresponding 95% confidence intervals, and the red dashed vertical line indicates the null value (OR = 1).

**Figure 4:**
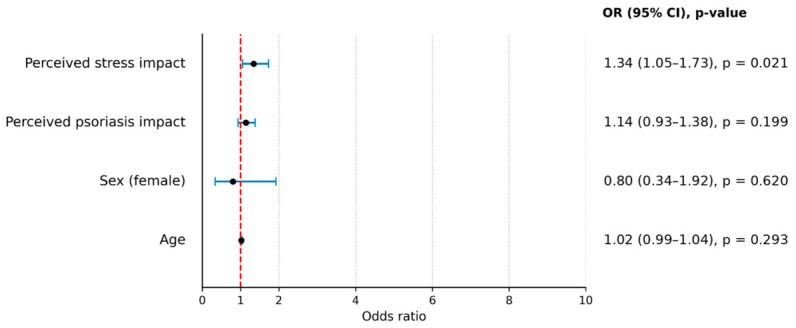
Forest plot showing adjusted odds ratios and 95% confidence intervals for factors associated with willingness to seek psychiatric support. Black dots represent the adjusted odds ratios, blue horizontal lines represent the corresponding 95% confidence intervals, and the red dashed vertical line indicates the null value (OR = 1).

Multivariable logistic regression was then used to assess whether these associations remained significant after adjustment for demographic and clinical factors; the corresponding models are presented in Table 10 and Table 11 and Figure 3 and Figure 4.

**Figure 5:**
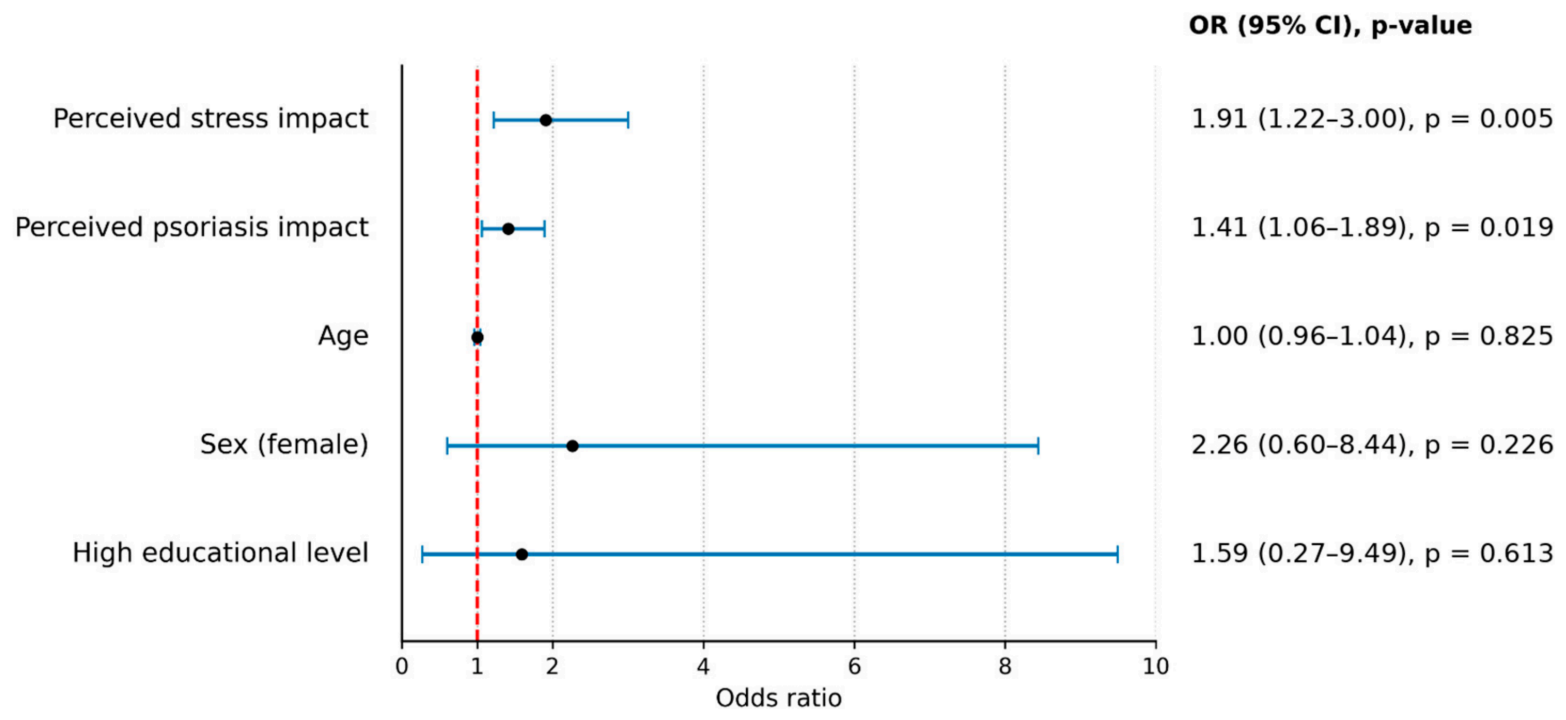
Forest plot showing adjusted odds ratios and 95% confidence intervals for factors associated with the perceived benefit of guidance by the dermatologist toward a psychologist or psychiatrist. Black dots represent the adjusted odds ratios, blue horizontal lines represent the corresponding 95% confidence intervals, and the red dashed vertical line indicates the null value (OR = 1).

Separate multivariable logistic regression models were performed to assess factors independently associated with the perceived benefit of guidance by the dermatologist toward a psychologist/psychiatrist (Table 12, Figure 5) and with previous psychological counseling or psychiatric treatment (Table 13).

### 3.4. Education-Related Outcomes

The association between educational level and patient-related outcomes is presented in Table 14.

### 3.5. Music Therapy-Specific Analyses

Given the central role of stress in the questionnaire framework, music therapy was analyzed separately as a complementary stress-oriented intervention.

A multivariable logistic regression model was constructed to identify factors independently associated with awareness of music therapy, as shown in Table 15.

Multivariable logistic regressions of clinical-demographic and music-related factors associated with willingness to try music therapy are presented in Table 16 and Table 17.

### 3.6. Secondary Analyses

#### 3.6.1. Psoriasis Type-Related Outcomes

The association between clinical forms of psoriasis and patient-related outcomes is presented in Table 18.

#### 3.6.2. Barriers to Mental Health Support

The association between stress level and barriers to mental health support is presented in Table 19.

## 4. Discussion

The present study shows that PsO is experienced by many patients as more than a skin disease alone. In our sample, both the perceived burden of PsO and the burden of psychological stress were clinically relevant, with mean scores of 5.81 ± 2.76 and 5.39 ± 2.24, respectively, and median scores of 6 [4–8] and 5 [4–7]. In addition, 86.3% of participants believed that psychological stress was related to the onset or worsening of their PsO.

These findings are consistent with previous literature showing that psychological stress is frequently perceived by patients as a relevant trigger for PsO onset or exacerbation [28]. Reported proportions vary widely between studies, from approximately 31% to 88%, depending on study design, stress definition, and method of assessment [28]. After adjustment for perceived PsO impact, age, sex, and educational level, perceived stress impact was the only variable independently associated with perceiving stress as related to PsO, with each one-point increase in stress burden nearly doubling the odds of this perception (OR 1.93, 95% CI 1.26–2.95; *p* = 0.002).

A clear difference emerged between attitudes toward psychological counseling and psychiatric consultation. Psychological counseling was the most accepted option, with 75.5% of participants endorsing it as a good idea if helpful, whereas psychiatric consultation was accepted by 48.0%. This contrast was also reflected in the pattern of non-acceptance: stigma-related responses were less frequent for psychological counseling than for psychiatric consultation (4.9% vs. 21.6%), and explicit lack of perceived need was also lower for psychological counseling (19.6% vs. 30.4%). Psychological counseling may be viewed as a more approachable and coping-oriented intervention, while psychiatric care may still be associated with greater stigma, more severe mental illness, or a higher threshold for perceived need. This difference is consistent with general mental health literature showing that help-seeking is influenced by stigma and treatment preferences [21,29,30]. In a systematic review including 144 studies and 90,189 participants, mental health-related stigma showed a small-to-moderate negative association with help-seeking, with a median effect size of d = −0.27 [21]. Treatment preference may also be relevant, as a meta-analytic review reported that approximately 75% of adults preferred psychological treatment over pharmacological treatment for psychiatric disorders [30].

Stress burden was one of the most consistent correlates of openness to support. Compared with patients in the low-stress group, those in the high-stress group were more likely to report willingness to see a psychologist (91.5% vs. 61.8%; OR 6.64, 95% CI 2.08–21.18; *p* < 0.001), willingness to see a psychiatrist (63.8% vs. 34.5%; OR 3.34, 95% CI 1.48–7.55; *p* = 0.003), willingness to try music therapy (70.2% vs. 41.8%; OR 3.28, 95% CI 1.44–7.47; *p* = 0.004), and previous psychological counseling or psychiatric treatment (34.0% vs. 12.7%; OR 3.54, 95% CI 1.31–9.59; *p* = 0.010).

Stigma-related barriers did not differ significantly between the low- and high-stress groups. For psychological support, any stigma-related barrier was reported by 7.3% of patients in the low-stress group and 2.1% in the high-stress group (*p* = 0.370; Cramér’s V = 0.07). For psychiatric support, the corresponding proportions were 20.0% and 23.4% (*p* = 0.810; Cramér’s V = 0.02). These findings suggest that higher perceived stress was not accompanied by higher reported shame or fear of social judgment, supporting the interpretation that openness to support among highly stressed patients may reflect greater perceived need rather than lower stigma alone.

The analyses based on continuous burden scores further support the association between subjective burden and receptiveness to supportive care. Patients who considered dermatologist guidance toward psychological or psychiatric support beneficial had higher median scores for both perceived PsO impact and perceived stress impact than those who did not (6 [5–9] vs. 3 [1–4] and 6 [5–7] vs. 3 [2–4], respectively; both *p* < 0.001), with moderate effect sizes (r = 0.47 and r = 0.52). A similar pattern was observed for willingness to see a psychologist, which was associated with higher PsO-impact and stress-impact scores, again with moderate effect sizes (r = 0.46 and r = 0.44; both *p* < 0.001).

A notable finding of the study is that psychological counseling and psychiatric consultation appeared to follow different patterns of acceptability. In multivariable analysis, willingness to seek psychological support was independently associated with perceived PsO impact (OR 1.43, 95% CI 1.11–1.85; *p* = 0.006), whereas willingness to seek psychiatric support was independently associated with perceived stress impact (OR 1.34, 95% CI 1.05–1.73; *p* = 0.021). This distinction is clinically relevant and suggests that psychological counseling may be perceived primarily as a coping-oriented response to the burden of the skin disease itself, while psychiatric consultation may be more closely linked to perceived stress burden or emotional distress.

The study’s findings also emphasize the dermatologist’s role as a potential entry point for psychodermatologic care. In our cohort, 80.4% of participants considered that guidance from the dermatologist toward psychological or psychiatric support would be beneficial. In multivariable analysis, this perception remained independently associated with both perceived stress burden (OR 1.91, 95% CI 1.22–3.00; *p* = 0.005) and perceived PsO burden (OR 1.41, 95% CI 1.06–1.89; *p* = 0.019). This suggests that patients with greater subjective burden may be particularly receptive to referral or guidance initiated within dermatologic care.

Perceived dermatologist involvement in stress-related care showed substantial variability, with a mean score of 5.93 ± 3.55 and a median of 6 [2–10], on a 0-to-10 scale. This suggests that, although most patients considered dermatologist guidance toward psychological or psychiatric support beneficial, stress-related care may not be addressed uniformly in routine dermatologic practice.

Actual prior use of psychological or psychiatric support was less frequent than declared openness to such interventions. In our sample, 22.5% of participants reported previous counseling or psychiatric treatment. This proportion is comparable to previous questionnaire-based data from Romanian patients with PsO, where 23% reported having consulted a psychologist and 7% a psychiatrist, and to prospective PsO screening data in which psychiatric treatment was accepted by 23.9% of patients after being recommended [17,18]. In our cohort, previous use of psychological or psychiatric support was independently associated with greater perceived stress burden (OR 1.57, 95% CI 1.13–2.18; *p* = 0.007) and younger age (OR 0.95, 95% CI 0.91–0.99; *p* = 0.009), suggesting that patients with higher stress burden and younger patients were more likely to have accessed mental health support.

Educational level also appeared to influence several support-related outcomes, particularly those related to music therapy. Participants with higher educational level more frequently reported willingness to see a psychologist (100.0% vs. 68.4%; *p* < 0.001), awareness of music therapy (78.3% vs. 41.8%; OR 5.02, 95% CI 1.69–14.88; *p* = 0.004), and willingness to try music therapy (78.3% vs. 48.1%; OR 3.88, 95% CI 1.31–11.49; *p* = 0.016). In the multivariable model, higher educational level remained independently associated with awareness of music therapy (OR 5.56, 95% CI 1.80–17.18; *p* = 0.003). Although these findings should be interpreted with some caution because of the relatively small number of participants with higher education, they suggest that educational level may influence familiarity with music therapy and openness to selected forms of supportive care.

In our study, willingness to try music therapy was reported by 54.9% of participants, despite awareness of music therapy being present in only 50.0% of respondents. This finding should be interpreted in the context of the high proportion of patients who reported listening to music for relaxation (86.3%), suggesting that music therapy may be acceptable even among patients with limited prior familiarity with this specific intervention. To our knowledge, there are no previous studies specifically evaluating awareness of, or willingness to try, music therapy among patients with PsO. However, the willingness observed in our cohort can be interpreted in the broader context of patients’ interest in complementary and supportive non-pharmacological approaches, as previous studies have reported the use of complementary approaches in 56.51–62% of patients with PsO [25,26,27].

A distinctive contribution of this study is the separate analysis of factors associated with music therapy awareness and willingness to try music therapy. In the clinical-demographic model, willingness to try music therapy was independently associated with high perceived stress impact (OR 3.01, 95% CI 1.28–7.09; *p* = 0.011) and higher educational level (OR 3.80, 95% CI 1.22–11.81; *p* = 0.021). In the music-related model, both listening to music for relaxation (OR 10.19, 95% CI 2.05–50.59; *p* = 0.005) and awareness of music therapy (OR 3.30, 95% CI 1.39–7.85; *p* = 0.007) were independently associated with willingness to try music therapy. These findings suggest that openness to music therapy is shaped by both subjective burden and prior familiarity with music as a relaxation strategy.

The reasons for refusing music therapy provide additional insight into patient attitudes. Among the 46 (45.1%) participants who were unwilling to try music therapy, the most common responses were “I can manage on my own” (32.6%) and “I do not think it would work” (28.3%). Preference for another form of psychotherapy was reported by 19.6%, while financial limitations (13.0%) and lack of time (6.5%) were less frequent. This pattern suggests that reluctance toward music therapy was driven mainly by perceived self-sufficiency and skepticism regarding efficacy, rather than by practical barriers alone.

Sex-related differences were present, but modest in effect size. Women reported higher perceived stress burden and greater perceived dermatologist involvement in stress-related care than men (Cohen’s d = 0.42 and 0.40, respectively). By contrast, the exploratory comparison between plaque PsO and other clinical forms did not show meaningful differences in perceived stress or support-related outcomes, with negligible to small effect sizes.

From a clinical perspective, these findings support the relevance of a psychodermatologic approach in PsO and emphasize the dermatologist’s role in identifying patients who may be receptive to supportive interventions. Asking about perceived stress burden, disease impact, and openness to different forms of support may help tailor referral strategies. Importantly, psychological counseling, psychiatric care, and music therapy should not be approached as a single construct, as they appeared to occupy distinct roles in patients’ help-seeking preferences. Although most associations were small to moderate in magnitude, they were consistent and clinically interpretable, suggesting that supportive care in PsO may be more effective when tailored not only to the level of perceived burden, but also to the specific form of help the patient is most ready to accept.

The study has several strengths. It examines multiple psychodermatologic dimensions within the same cohort, combines descriptive analyses with multivariable regression models, and evaluates music therapy as a distinct supportive option rather than limiting the analysis to conventional psychological or psychiatric care. At the same time, several limitations should be acknowledged. The cross-sectional design does not allow conclusions about causality. The sample was relatively small and recruited from a single university dermatology center, which may limit generalizability. The absence of an a priori sample size calculation and the relatively small sample may have limited the precision of the estimates, particularly in the multivariable analyses, which should therefore be considered exploratory. The questionnaire was original and not previously validated, and the study relied on self-reported perceptions rather than standardized psychometric or disease-severity instruments. Formal content and construct validation and test–retest reliability assessment were not performed. In particular, perceived stress impact was assessed using a single numerical rating item intended as a brief, exploratory measure suitable for routine clinical administration, rather than as a comprehensive or diagnostic assessment of psychological stress. Consequently, the findings should be interpreted as exploratory associations based on patient-reported perceptions, and direct comparisons with studies using validated multidimensional stress instruments should be made with caution. The threshold used to categorize low and high perceived stress impact was exploratory and not clinically validated. Although analyses using the continuous score were also performed, dichotomization may have resulted in some loss of information and should be considered when interpreting the grouped analyses. PsO duration, objective disease severity measures, and formally diagnosed psychiatric comorbidities were not systematically collected. Consequently, the analyses could not account for the potential influence of disease chronicity, clinical severity, or psychiatric history on perceived burden and openness to supportive care. Because participants were recruited from a single Romanian center, the findings may not reflect attitudes toward music therapy in populations with different cultural backgrounds or musical traditions. The high proportion of patients perceiving psychological stress as a relevant trigger for PsO onset or exacerbation may partly reflect the study setting and period. Patients recruited from a university dermatology center may have greater disease complexity. Moreover, data were collected between 2023 and 2025, in a period marked by post-pandemic psychological strain and regional geopolitical instability, factors that may have amplified perceived stress and its attribution to PsO. Current and previous psoriasis treatments, treatment duration, adherence, and therapeutic response were not systematically collected. In addition, previous psychological counseling or psychiatric treatment was assessed only as a combined dichotomous variable, without information regarding the specific intervention, indication, timing, or current treatment status. Consequently, the potential influence of previous or ongoing treatment on perceived burden and openness to supportive care could not be evaluated. Finally, the study assessed attitudes and previous use of supportive care, but not subsequent referral uptake, adherence to interventions, or clinical outcomes after psychological, psychiatric, or music therapy-based support.

Future research should include larger, multicenter, and culturally diverse samples and should use longitudinal or interventional designs to clarify the temporal relationships between perceived stress, PsO burden, and openness to supportive care. Validated psychometric instruments and standardized measures of PsO severity and quality of life should be incorporated. Follow-up assessments should evaluate whether expressed willingness translates into referral uptake, intervention adherence, and changes in psychological and dermatologic outcomes. For music therapy specifically, future studies should use clearly defined, preference-informed protocols and consider individual musical preferences, cultural associations with music, and participants’ previous musical experiences when evaluating acceptability and therapeutic response.

## 5. Conclusions

PsO was associated, in this cohort, with a substantial psychodermatologic burden, and most patients perceived psychological stress as relevant to the onset or worsening of their disease. Greater perceived stress and disease burden were associated with increased openness to supportive interventions, although the associated factors differed according to the type of support considered. Psychological counseling was more acceptable than psychiatric consultation, while music therapy emerged as a potentially acceptable complementary option, particularly among patients with higher stress burden, higher educational level, habitual music listening, and awareness of music therapy. These findings support the value of an individualized psychodermatologic approach in routine PsO care, tailored not only to perceived burden, but also to the form of support the patient is most ready to accept. Given the exploratory, cross-sectional, single-center design and the use of an original non-validated questionnaire, these findings require confirmation in larger multicenter and longitudinal studies using validated psychometric and clinical severity instruments.

## Figures and Tables

**Table 1:** Sociodemographic and clinical characteristics of the study population (*n* = 102).

Variable	Category/Statistic	*n* (%)	Value
Age	Mean ± SD		47.1 ± 15.3
	Median (IQR)		48.5 (34.0–60.0)
	Range		19–81
Sex	Female	55 (53.9%)	
	Male	47 (46.1%)	
Educational level	≤10 grades	57 (55.9%)	
	High school	22 (21.6%)	
	Bachelor’s degree	18 (17.6%)	
	Master’s degree	3 (2.9%)	
	PhD	2 (2.0%)	
Clinical type of psoriasis	Plaque psoriasis	77 (75.5%)	
	Pustular psoriasis	11 (10.8%)	
	Guttate psoriasis	6 (5.9%)	
	Inverse psoriasis	4 (3.9%)	
	Erythrodermic psoriasis	3 (2.9%)	
	Nail psoriasis	1 (1.0%)	

**Table 2:** Descriptive analysis of the main psychodermatologic variables.

Variable	Category/Statistic	*n* (%)	Value
Perceived impact of psoriasis on daily life	Mean ± SD		5.81 ± 2.76
	Median (IQR)		6 (4–8)
	Range		0–10
Perceived impact of psychological stress on daily life	Mean ± SD		5.39 ± 2.24
	Median (IQR)		5 (4–7)
	Range		0–10
Perceived relationship between stress and psoriasis	Yes	88 (86.3%)	
	No	14 (13.7%)	

**Table 3:** Descriptive statistics for dermatologist-related psychodermatologic care and previous use of mental health support.

Variable	Category/Statistic	Value
Perceived dermatologist involvement in stress-related care	Mean ± SD	5.93 ± 3.55
	Median (IQR)	6 (2–10)
	Range	0–10
Perceived benefit of guidance by the dermatologist toward a psychologist/psychiatrist	Yes	82 (80.4%)
	No	20 (19.6%)
Previous psychological counseling or psychiatric treatment	Yes	23 (22.5%)
	No	79 (77.5%)

**Table 4:** Attitudes toward psychological counseling, psychiatric care, and music therapy. * Percentages for reasons for refusing music therapy were calculated among patients who answered No to willingness to try music therapy (*n* = 46).

Variable	Category	*n*	%
Opinion on psychological counseling	It seems like a good idea if it can help me	77	75.5
	I would feel ashamed to seek psychological counseling	3	2.9
	I am afraid that relatives or friends would judge me if I went to a psychologist	2	2.0
	I do not need it	20	19.6
Opinion on psychiatric consultation/treatment	It seems like a good idea if it can help me	49	48.0
	I would feel ashamed to consult a psychiatrist	12	11.8
	I am afraid that relatives or friends would judge me if I went to a psychiatrist	10	9.8
	I do not need it	31	30.4
Listening to music for relaxation	Yes	88	86.3
	No	14	13.7
Awareness of music therapy	Yes	51	50.0
	No	51	50.0
Willingness to try music therapy	Yes	56	54.9
	No	46	45.1
Reasons for refusing music therapy *	I do not have enough time	3	6.5
	I do not have sufficient financial resources	6	13.0
	I do not think it would work	13	28.3
	I would prefer another form of psychotherapy	9	19.6
	I can manage on my own	15	32.6

**Table 5:** Sex-related differences in dichotomous psychodermatologic variables (* OR expressed for men vs. women).

Variable	Women, *n* (%)	Men, *n* (%)	OR (95% CI) *	*p*-Value
Willingness to try music therapy	34/55 (61.8)	22/47 (46.8)	0.54 (0.25–1.20)	0.163
Awareness of music therapy	31/55 (56.4)	20/47 (42.6)	0.57 (0.26–1.26)	0.233
Listening to music for relaxation	47/55 (85.5)	41/47 (87.2)	1.16 (0.37–3.63)	1.000
Previous psychological counseling or psychiatric treatment	14/55 (25.5)	9/47 (19.1)	0.69 (0.27–1.79)	0.485
Perceived benefit of guidance by the dermatologist toward a psychologist/psychiatrist	48/55 (87.3)	34/47 (72.3)	0.38 (0.14–1.06)	0.080

**Table 6:** Sex-related differences in continuous variables.

Variable	Women (*n* = 55) Mean ± SD	Men (*n* = 47) Mean ± SD	Mean Difference	*p*-Value	Cohen’s d
Age	45.82 ± 14.61	48.55 ± 16.14	−2.73	0.371	0.18
Perceived impact of psoriasis on daily life (0–10)	6.20 ± 2.67	5.36 ± 2.83	0.84	0.127	0.31
Perceived impact of psychological stress on daily life (0–10)	5.82 ± 2.27	4.89 ± 2.12	0.92	0.037	0.42
Perceived dermatologist involvement in stress-related care (0–10)	6.58 ± 3.47	5.17 ± 3.52	1.41	0.045	0.40

**Table 7:** Multivariable logistic regression analysis of factors associated with the perceived relationship between stress and psoriasis.

Variable	β	SE	OR (95% CI)	*p*-Value
Perceived stress impact	0.66	0.22	1.93 (1.26–2.95)	0.002
Perceived psoriasis impact	−0.13	0.13	0.88 (0.68–1.15)	0.348
Age	0.00	0.02	1.00 (0.96–1.04)	0.913
Sex (female)	0.14	0.64	1.15 (0.33–4.05)	0.824
High educational level	−0.50	0.79	0.60 (0.13–2.81)	0.521

**Table 8:** Help-seeking and support-related outcomes according to stress level.

Outcome	Low Stress ≤ 5 (*n* = 55)	High Stress > 5 (*n* = 47)	OR (95% CI)	*p*-Value	Cramér’s V
Willingness to see a psychologist	34 (61.8%)	43 (91.5%)	6.64 (2.08–21.18)	<0.001	0.34
Willingness to see a psychiatrist	19 (34.5%)	30 (63.8%)	3.34 (1.48–7.55)	0.003	0.29
Willingness to try music therapy	23 (41.8%)	33 (70.2%)	3.28 (1.44–7.47)	0.004	0.26
Previous psychological counseling or psychiatric treatment	7 (12.7%)	16 (34.0%)	3.54 (1.31–9.59)	0.010	0.23

**Table 9:** Association between perceived psoriasis impact, perceived stress impact, and help-seeking-related outcomes.

Variable	Perceived Psoriasis Impact, Yes (Median [IQR])	Perceived Psoriasis Impact, No (Median [IQR])	*p*-Value	r	Perceived Stress Impact, Yes (Median [IQR])	Perceived Stress Impact, No (Median [IQR])	*p*-Value	r
Perceived benefit of guidance by the dermatologist toward a psychologist/psychiatrist	6 [5–9]	3 [1–4]	<0.001	0.47	6 [5–7]	3 [2–4]	<0.001	0.52
Previous psychological counseling or psychiatric treatment	6 [4–9]	6 [3–7]	0.098	0.16	7 [5–8]	5 [3.5–6.5]	<0.001	0.33
Willingness to see a psychologist	6 [5–9]	3 [1–5]	<0.001	0.46	6 [5–7]	3 [2–5]	<0.001	0.44
Willingness to see a psychiatrist	6 [5–9]	5 [3–7]	0.002	0.30	6 [5–8]	5 [3–6]	<0.001	0.37

**Table 10:** Multivariable logistic regression for willingness to seek psychological support.

Variable	β	SE	OR (95% CI)	*p*-Value
Perceived psoriasis impact	0.36	0.13	1.43 (1.11–1.85)	0.006
Perceived stress impact	0.32	0.17	1.38 (0.99–1.92)	0.059
Sex (female)	0.52	0.56	1.68 (0.56–5.02)	0.352
Age	−0.003	0.017	1.00 (0.96–1.03)	0.847

**Table 11:** Multivariable logistic regression for willingness to seek psychiatric support.

Variable	β	SE	OR (95% CI)	*p*-Value
Perceived stress impact	0.29	0.13	1.34 (1.05–1.73)	0.021
Perceived psoriasis impact	0.13	0.10	1.14 (0.93–1.38)	0.199
Sex (female)	−0.22	0.44	0.80 (0.34–1.92)	0.620
Age	0.015	0.014	1.02 (0.99–1.04)	0.293

**Table 12:** Multivariable logistic regression for perceived benefit of guidance by the dermatologist toward a psychologist/psychiatrist.

Variable	β	SE	OR (95% CI)	*p*-Value
Perceived stress impact	0.65	0.23	1.91 (1.22–3.00)	0.005
Perceived psoriasis impact	0.35	0.15	1.41 (1.06–1.89)	0.019
Age	−0.00	0.02	1.00 (0.96–1.04)	0.825
Sex (female)	0.81	0.67	2.26 (0.60–8.44)	0.226
High educational level	0.46	0.91	1.59 (0.27–9.49)	0.613

**Table 13:** Multivariable logistic regression for previous psychological counseling or psychiatric treatment.

Variable	β	SE	OR (95% CI)	*p*-Value
Perceived stress impact	0.45	0.17	1.57 (1.13–2.18)	0.007
Perceived psoriasis impact	−0.02	0.13	0.98 (0.76–1.26)	0.863
Age	−0.05	0.02	0.95 (0.91–0.99)	0.009
Sex (female)	−0.06	0.55	0.94 (0.32–2.77)	0.909
High educational level	0.57	0.57	1.76 (0.58–5.37)	0.321

**Table 14:** Association between educational level and patient-related outcomes. Odds ratio and 95% confidence interval could not be estimated for willingness to see a psychologist due to a zero cell count (all patients with high educational level expressed willingness).

Outcome	Low Education *n* (%)	High Education *n* (%)	OR (95% CI)	*p*-Value	Cramér’s V
Willingness to see a psychologist	54 (68.4%)	23 (100%)	—	<0.001	0.31
Willingness to see a psychiatrist	36 (45.6%)	13 (56.5%)	1.55 (0.61–3.96)	0.478	0.09
Listening to music	66 (83.5%)	22 (95.7%)	4.33 (0.54–35.05)	0.182	0.15
Awareness of music therapy	33 (41.8%)	18 (78.3%)	5.02 (1.69–14.88)	0.004	0.30
Willingness to try music therapy	38 (48.1%)	18 (78.3%)	3.88 (1.31–11.49)	0.016	0.25
Previous psychological counseling or psychiatric treatment	15 (19.0%)	8 (34.8%)	2.28 (0.82–6.35)	0.155	0.16
Perceived benefit of guidance by the dermatologist toward a psychologist/psychiatrist	61 (77.2%)	21 (91.3%)	3.10 (0.66–14.49)	0.231	0.15

**Table 15:** Multivariable logistic regression analysis of factors associated with awareness of music therapy.

Variable	β	SE	OR (95% CI)	*p*-Value
High educational level	1.72	0.58	5.56 (1.80–17.18)	0.003
Age	0.02	0.01	1.02 (0.99–1.05)	0.228
Sex (female)	0.63	0.43	1.88 (0.81–4.37)	0.140
Listening to music	0.05	0.61	1.05 (0.31–3.49)	0.940

**Table 16:** Multivariable logistic regression of clinical-demographic factors associated with willingness to try music therapy.

Variable	β	SE	OR (95% CI)	*p*-Value
High perceived stress impact (>5 vs. ≤5)	1.10	0.44	3.01 (1.28–7.09)	0.011
High educational level	1.33	0.58	3.80 (1.22–11.81)	0.021
Sex (female)	0.56	0.44	1.75 (0.75–4.11)	0.199
Age	0.01	0.01	1.01 (0.98–1.04)	0.559
Constant	−1.25	0.81	0.29 (0.06–1.40)	0.122

**Table 17:** Multivariable logistic regression of music-related factors associated with willingness to try music therapy.

Variable	β	SE	OR (95% CI)	*p*-Value
Music listening	2.32	0.82	10.19 (2.05–50.59)	0.005
Awareness of music therapy	1.19	0.44	3.30 (1.39–7.85)	0.007
Constant	−2.43	0.83	0.09 (0.02–0.45)	0.003

**Table 18:** Association between clinical form of psoriasis and patient-related outcomes.

Outcome	Plaque Psoriasis (*n* = 77)	Other Forms of Psoriasis (*n* = 25)	*p*-Value	Effect Size
Perceived stress (0–10)	5.38 ± 2.28	5.44 ± 2.14	0.830	Cohen’s d = 0.03
Willingness to try music therapy	43 (55.8%)	13 (52.0%)	0.917	Cramér’s V = 0.01
Willingness to see a psychologist	61 (79.2%)	16 (64.0%)	0.204	Cramér’s V = 0.13
Willingness to see a psychiatrist	38 (49.4%)	11 (44.0%)	0.814	Cramér’s V = 0.02

**Table 19:** Association between stress level and barriers to mental health support.

Support Type	Barrier	Low Stress ≤ 5 (*n* = 55)	High Stress > 5 (*n* = 47)	*p*-Value	Cramér’s V
Psychological support	Shame	2 (3.6%)	1 (2.1%)	1.000	0.00
	Fear of social judgment	2 (3.6%)	0 (0%)	0.498	0.06
	Any barrier	4 (7.3%)	1 (2.1%)	0.370	0.07
Psychiatric support	Shame	7 (12.7%)	5 (10.6%)	1.000	0.00
	Fear of social judgment	4 (7.3%)	6 (12.8%)	0.507	0.06
	Any barrier	11 (20.0%)	11 (23.4%)	0.810	0.02

## Data Availability

The data presented in this study are available from the corresponding authors upon reasonable request. The data are not publicly available due to privacy and ethical restrictions.

## Supplementary Materials

The following supporting information can be downloaded at: https://www.mdpi.com/article/10.3390/jcm15155984/s1, File S1: Study questionnaire.

## Abbreviations

The following abbreviations are used in this manuscript:

CI	Confidence interval
IQR	Interquartile range
OR	Odds ratio
PsO	Psoriasis
SD	Standard deviation
SE	Standard error
