# Supplementary material for: Psychodermatologic Dimensions of Psoriasis: Perceived Burden, Help-Seeking Behavior and Openness to Supportive Care
Source: J Clin Med. 2026 Jul 31;15(15):5984. doi: 10.3390/jcm15155984 (PMC13466355; doi:10.3390/jcm15155984)
Supplement: Supplementary file 1 [file jcm-15-05984-s001.zip › jcm-4430991-supplementary.pdf]

Supplementary File S1

Study Questionnaire

Psoriasis and Music Therapy Questionnaire

Note: The questionnaire was originally administered in Romanian. The version below represents an English translation prepared for supplementary material.

Estimated completion time: 3 minutes

The study was conducted in accordance with Law No. 206/2004 (updated until 2016) on good conduct in scientific research, technological development and innovation; Law No. 319/2003 on the Statute of Research and Development Personnel; and Regulation (EU) 2016/679 of 27 April 2016 on the protection of natural persons with regard to the processing of personal data and on the free movement of such data, repealing Directive 95/46/EC (General Data Protection Regulation).

1. What is your sex?

☐ Male
☐ Female

2. What is your age?

____

3. What is the highest level of education you have completed?

☐ Less than or equal to 10 grades
☐ High school diploma
☐ Bachelor's degree
☐ Master's degree
☐ PhD

4. Which form of psoriasis has your dermatologist diagnosed you with?

☐ Plaque psoriasis
☐ Pustular psoriasis
☐ Guttate psoriasis
☐ Inverse psoriasis
☐ Erythrodermic psoriasis
☐ Nail psoriasis

5. On a scale from 0 to 10, how much does psoriasis affect your life (average over the last 3 months)?

(0 = not at all; 10 = maximum impact)

0	1	2	3	4	5	6	7	8	9	10

6. On a scale from 0 to 10, how much does psychological stress affect your daily life (average over the last 3 months)?

(0 = not at all; 10 = maximum stress)

0	1	2	3	4	5	6	7	8	9	10

7. Do you believe that psychological stress is related to the onset or worsening of your psoriasis?

☐ Yes

☐ No

8. In the context of your skin condition, do you consider that your dermatologist addressed the issue of psychological stress?

(0 = not at all; 10 = yes, this aspect was fully addressed)

0	1	2	3	4	5	6	7	8	9	10

9. Do you consider that it would be beneficial for your dermatologist to counsel you and, if appropriate, guide you toward a psychologist/psychiatrist for the management of psychological stress?

☐ Yes

☐ No

10. Have you ever received psychological counseling or psychiatric treatment, regardless of the reason?

☐ Yes

☐ No

11. What is your opinion regarding psychological counseling?

☐ It seems like a good idea if it can help me

☐ I would feel ashamed to seek psychological counseling

☐ I am afraid that my relatives or friends would judge me if I went to a psychologist

☐ I do not need it

12. What is your opinion regarding psychiatric consultation and treatment?

☐ It seems like a good idea if it can help me

☐ I would feel ashamed to consult a psychiatrist

☐ I am afraid that my relatives or friends would judge me if I went to a psychiatrist

☐ I do not need it

13. During your free time or at work, do you listen to music for relaxation?

☐ Yes

☐ No

14. Have you heard of music therapy (therapy through music)?

☐ Yes

☐ No

15. Would you like to receive music therapy for the purpose of reducing psychological stress and possibly, subsequently, improving psoriasis lesions, considering that psychological stress is closely related to this skin condition?

Music therapy can be defined as a non-verbal therapy that uses sound and music, in all its forms, to promote, maintain, and restore mental, physical, emotional, and spiritual health.

☐ Yes

☐ No

16. If not, what would be your reasons?

☐ I do not have enough time
☐ I do not have sufficient financial resources for this
☐ I do not think it would work
☐ I would prefer another form of psychotherapy
☐ I can manage on my own

Consent statement

I declare that I have been informed regarding data protection. I agree that the data obtained from this study may be published, and I understand that my confidentiality will be protected at all times.

Date	Full Name	Signature
